# Proceedings of the Medical Convention of Ohio, Held at Cincinnati on the 16th, 17th, 18th, 19th, and 20th, of May, 1842

**Published:** 1843-06

**Authors:** 


Art. IX.—*Proceedings of the Medical Convention of Ohio, held at Cincinnati on the* 16*th*, 17*th*, 18*th*, 19*th*, *and* 20*th*, *of May*, 1842. *With selections from papers read before that body*. Cincinnati, R. P. Brooks, 1842. pp. 51.

We observed in the Journal for April that, of the various papers presented to the Convention, but two were published. The first and longest of these, is entitled "A History of the Topography, Climate and Diseases of the County of Scioto, from its settlement to the present time." In the outset of this paper, the author, Dr. Hempstead, pays a well deserved tribute to the hardy and enterprising men, who led the way in the settlement of the country, and he acknowledges his indebtedness to them for much valuable information regarding the diseases of the early inhabitants. Doubtless much might still be gleaned in this way—as these individuals, otherwise of acute and retentive minds, were oftentimes compelled to be their own physicians.

Dr. H. thus describes the region of country to which his observations refer:

"The county of Scioto is situated upon the southern border of the state, at the confluence of the Scioto and Ohio rivers, and in Lat. 38° 38″ N., and 82° 56″ W., extending North about twenty miles, and including the table land for twenty miles East and West on either side of the Scioto river. The valley proper is based on a bed of shale, which may be seen cropping out a few miles below Portsmouth, and disappears not far from the western line of the county, near the great western limestone deposit. The table land is here elevated from three to five hundred feet above the surface of the valley. It is gently undulating, but as it approaches the Scioto it becomes very precipitous, and, in most places, incapable of cultivation. The tributaries of the Scioto, which arise in this region, are very rapid, highly charged with lime, and subject to great alternations from the most rapid and violent torrents, to the most perfect destitution of all moisture. On the east side of the valley, the surface is not so high by two hundred feet. It also rises less abruptly than on the west. Still, it is undulating, and affords fine grazing and arable farms. The water courses, however, are not so numerous as they are on the opposite side of the river. Iron, coal, and saliferous rock are found in this locality, which is bounded on the east by the buhr-stone deposit. Out of the valley proper, no ponds or stagnant waters are found, the vegetation is less luxuriant, and of a more durable and ligneous character, than that found in the alluvions immediately bounding the Scioto. Between the low bottoms and the river hills, sandy bluffs occasionally occur, composed principally of coarse gravel and sand, with a very thin vegetable mould, soon exhausted by cultivation, and when the soil becomes impoverished, it is not easily renewed, especially as these bluffs are too high to be benefitted by the spring floods, which annually inundate and enrich the low grounds. Upon these bluffs, elevated from ten to forty feet above the highest floods, are found those monuments of a race long since departed, but still exhibiting, by their works, the strongest proof of having been a populous, an industrious, and a talented people. These evidences of a laborious, intelligent, and, to some extent, a civilized community, are to be found, at short intervals, upon either side of the Ohio and Scioto rivers, occasionally extending into the valley, but occupying ground above all ordinary floods. The soil west of the Scioto is good, containing a portion of sand, and possessing the characteristics of a calcareous deposit. Elevated from four to six hundred feet above the valley, it descends towards the east, exposing the limestone, waverly sandstone, and slaty argillacious rock, which last underlies the valley proper. From this point the surface rises some three hundred feet, changing its character, and becoming a pure clay. Although more broken by hills, and less suited to agriculture, it is rich in mineral wealth.

"The valley of the Scioto, from two to five miles in width, possesses a soil unsurpassed in fertility and durability by any other; being composed of the debris and washings of the uplands, with a large mixture of decayed vegetable matter deposited by the spring floods which annually inundate it.

"The southern border of this locality, comprising the valley of the Ohio, differs but little from the alluvions of the Scioto; since the low bottoms of the former, which are frequently inundated, possess all the fertility and durability of the latter, while the high or "second bottoms," which are mostly argillaceous, are less productive, being destitute of that rich arenaceous deposit, which annually renews and ameliorates those less elevated. The table land of the region now under consideration is covered with all the varieties of the oak, except the highest points, which contain groves of pine. The slopes connecting the bottoms with the upland exhibit a general mixture of western trees, including the locust, pawpaw, sugar tree, &c., while the sycamore, cottonwood, black walnut, mulberry, maple, and elm, occupy the lower portions of the valley. There is not much undergrowth, except in the low valley, which consists of a luxuriant production of annual plants, that are constantly decomposing and enriching the soil upon which they grow.* The Ohio interval produces beech, hickory, and maple, with sycamore and elm on the margin of the stream."

The inhabitants of this region are supplied with water from the Ohio and Scioto rivers, and their tributaries, and from wells and springs, which vary on different sides of the valley.

"On the east side of the valley, fine springs of soft, wholesome, and pleasant water, like that of the Ohio river, above its junction with the Scioto, are found in abundance, free from iron or other minerals. The wells in their vicinity are of the same character, while the springs and wells west partake of the character of the country in which they are situated, being, like the water of the Scioto, strongly impregnated with calcareous matter."

He mentions the following singular circumstance, which we do not remember to have seen noticed elsewhere.

"A singular and interesting circumstance, connected with the ancient works in Portsmouth and its vicinity, affecting some of its wells, deserves here to be mentioned, and may throw some light upon the uses for which at least some of those embankments were constructed. North of the east end of the town, on a sandy elevation of some two hundred feet above the Ohio bottom, the construction of which has much the appearance of being artificial, are found two parallel embankments, which descend to the *sand bluffs* before mentioned, and then pass down to the Ohio bottom, where it is elevated from three to seven feet above the highest known flood. These embankments, continuing on the same elevation, enter the corporation at its east end, and pass through the north part of the town to the Scioto river, where they are

* The writer, some ten or fifteen years since, frequently passed through this undergrowth. When on horseback, he was unable to reach, with a common riding-whip, the top of a growth of vegetation standing so thick over the uncultivated portions of the valley as entirely to exclude the sun.

lost, evidently from the encroachments of that stream, for they again appear some distance below, and terminate on the bank of the Ohio, a short distance above the *present* mouth of the Scioto. Upon, or very near to these embankments, which vary from three feet in height to almost entire obliteration, a number of wells have been sunk to the depth of sixteen feet, furnishing an abundant supply of water, as pure as the mountain rill, being filtered through the fine sand, which is deposited by the Ohio when it overflows its banks. The wells which are located four or five rods north or south of these embankments pass through the natural clay of the bottom, from fifty to sixty feet, before a full supply of water can be obtained. It would seem that two intrenchments had been excavated in the clayey soil at least sixteen feet deep, and then filled with sand. The compact character of the clay confines the water, while the sand supports the clay walls, and permits the water to filter through. Whether this water is conducted from the hills, or collected between the embankments from rains, has not yet been satisfactorily ascertained. In its sensible properties, it is like that of the springs at the foot of the hills, upon which the embankments commence. Half a mile east of the town are some mounds, and an elevation, of the same sand, comprising about two acres, including the embankments. This sandy elevation has a number of springs around its margin, some of which rise to the surface; others are found in three or four feet excavation, a thing unusual on the Ohio bottoms. The writer has a spring in his cellar, from the same source, (although he is located more than thirty rods from the embankment), which rises to within four feet of the surface. It is two feet deep, and occasionally disappears in very extreme dry weather; while the wells, as before stated, never sink more than six feet below the surface, and frequently run over the top."

Mineral and medicinal springs abound.

"Those of the east side of the valley contain salt and iron, petroleum or bituminous oil; and one deposits, for two or three rods from its origin, a substance as white as snow, supposed to be magnesia, but more probably sulphate of lime. The chalybeate springs hold iron in such minute divisions as to be well suited to those cases of excitable debility which frequently occur and are often aggravated by any of the pharmacological forms of this tonic. These springs have been resorted to with much and decided benefit; they are generally situated in a mountain region, high, healthy, and among the furnaces, where novelty, exercise, and amusement, are not wanting. The springs of the western or limestone region are occasionally charged with sulphur, soda, magnesia, iron, and other salts. On the waters of Brush creek, about four or five miles from the Scioto valley, around the margin of an elevated portion of glady country, a number of medicinal springs are found, containing a variety of salts, and differing sowewhat in character from each other. As these are situated in a region unsurpassed for romantic scenery, above miasmatic influence, and possessing the finest hunting and fishing ground in the State, they may, at no distant period, become a desirable resort for health and amusement."

Sulphuret and sulphate of iron are found in great quantity on the west side of the valley near the Ohio. In the summer of 1839, he witnessed an interesting phenomena. "The rocky bed of the creek had been dry for some time and exposed for many days to a temperature above 90°, when a number of explosions occurred from the expansion and eruption of large masses of very pure pyrites, imbedded in the solid rock, leaving excavations indicating the boulder of sulphuret of iron to have been from twelve to eighteen inches in diameter."

That magnificent public work, the Ohio and Erie canal, passes through this county. When it was first projected, fears were entertained that the ponds and pools created by it would exert an unfavorable influence upon the health of the inhabitants; but these fears proved groundless.

"A careful observation for the last nine years in the vicinity of this improvement, has not detected any additional malarious influence, any increase of disease, or any new ailment affecting in any way the health of the inhabitants, excepting during the autumn when the excavation was going forward, at which time it was visited with an unusual amount of disease. This latter circumstance was also peculiarly apparent in 1840 and 1841, upon that part of "the Portsmouth and Columbus turnpike" which runs through the same county, especially where excavations and embankments were progressing during the fall months. In consequence of accidents, or to make repairs, the water of the canal has been repeatedly drawn off at the most unfavorable season, exposing an extensive slimy and foul surface to the action of an autumnal sun, until all moisture has been dissipated, and yet no deleterious influence has been the result. So far from this being the case, a single incident in 1837 goes to show an opposite effect. The ordinary diseases of the summer had been rather prevalent in the immediate vicinity of the canal, and a considerable number were then sick. On the 8th of September the water was drawn off, and the writer is confident that six new cases did not occur in that vicinity, for the balance of the season."

A portion of this region is subject to inundations from the Ohio and Scioto rivers; these were more frequent from 1795 to 1820, than they have been since. The differences in the diseases of the river bottoms, and of the uplands covered with forests, are striking; the purely miasmatic being confined to the former or inundated portions, while a different class occupies the higher regions.

The following picture of the habitations and manners of the early settlers, is true to the life.

"The residences of the early settlers were, as at the present day, on the banks of the streams, or on the slopes connecting the bottoms with the upland. They were built of logs, and well ventilated, not by windows and doors, but by the interstices between the logs. They were also occasionally washed by rains dashing through the imperfect covering. These walls, with an opening in the roof for smoke, and an earthen or puncheon floor, completed the domiciliary comforts of the early pioneer. Their scanty and thin clothing protected them still less against "the peltings of the pitiless storm," than their open and uncomfortable cabins. It was no uncommon occurrence for an individual to pass the winters of that period, with no other outer garments than the pataloons and hunting shirt, and these of the thinnest fabric of cotton and wool, the bare thoughts of which at the present day would cause a shudder at almost any season. The diet of this hardy race was in keeping with their other comforts. The fruits of the chase, a few vegetables in their season, and coarse cakes, prepared from meal ground upon the invaluable, ever revolving, and never idle *hand-mill*, constituted their whole bill of fare. Their amusements corresponded with their hardy character. These consisted in aiding a neighbor to husk his corn, to raise a cabin, to roll his logs; or among the females, to quilt a quilt; at which as well as at most other assemblies a bottle of "*Monongahela*" was a universal accompaniment. After the work was done, shooting at a mark, pitching quoits, wrestling, jumping, dancing, and by way of accommodation an occasional game of "rough and tumble," filled up the balance of the day and part of the night."

The climate of this region, like all in this great valley, perhaps we might say all on the western shore of the Atlantic, is subject to great and sudden alterations.

"The changes are very sudden and rapid, not unfrequently varying the temperature forty degrees in twelve hours, and instances have been noticed in which a change of fifty degrees has occurred in eight hours. For want of a series of observations sufficiently extensive no fixed character can, as yet, be given to our climate, although this desideratum will no doubt be soon obtained if the regulations established by the Surgeon General of the army be faithfully and carefully complied with at the several military posts. A sufficiency, however, is known to establish the fact that for some years past our seasons have been changing. Previous to 1820 the winters were not so cold, nor the summer so warm as they have been since that period, and particularly since 1830, which was the first time for some years that the thermometer was below zero. From the settlement of the county in 1795, the winters were mild, with a few days of severe weather, while the summers were cool, with an occasional day of elevated temperature. And although there has been manifestly a change, as will appear by the abstracts accompanying this paper, yet the mean temperature for the year was greater before than since the period mentioned. The mean temperature of the six years preceding 1830 being 55–45, while the six years since were but 54–75. So far as any statistics of the weather can be obtained, the mean temperature of the year, or of the hot or cold months, has not materially differed since the settlement of the county. The same remark will apply to the amount of rain; but the fogs, winds and violent storms, have undergone a very great change. From the first settlement of the county to 1820, the fogs usually commenced early in July, were very regular in their daily appearance, suspended only for a night or two previous to rain. They generally were observable as early as 9 o'clock, P. M., and frequently continued till the same hour the next morning, and occasionally till 11 o'clock, being throughout very dense and loaded with moisture. It was not unusual to see, during a dense fog, the water fall from the leaves of the trees like a shower of rain. These characteristics of the fogs of that period did not undergo much sensible change until '26 or '27, since which they fall later in the night, frequenly not till near day-light in the morning, and are with few exceptions dissipated by 8 o'clock A. M. The amount of moisture has greatly decreased, and it is not unusual for fogs to disappear during the summer and autumn for nights in succession, or to be seen only in the valleys and on the immediate neighboring hills. Their effects, whatever they may be, are not of course observable, upon the higher and more elevated regions. The prevailing winds of the first twenty-five years were westerly. In 1838, an ordinary observer must have discovered that they were decidedly on the decrease. In 1838 the easterly were to the westerly as one to two, in '39 and '40 nearly balanced, and in '41 were decidedly the prevailing wind of the year. The writer is well assured, from the authority of others, and partly from his own observation that from the year 1800 to 1810 no fall of rain was attended by an easterly wind. From that period to 1820 this wind was steadily and gradually on the increase, and from '21 onward has been observable, occasionally throughout each year. For the last twenty years those violent tornadoes, accompanied by thunder, which formerly swept so frequently through our State, have become less frequent and violent, and for the last few years have nearly disappeared. Hail, which attended them particularly in the month of June, is in this region an uncommon occurrence; very few storms of this kind having been observed in the last ten years. Electrical phenomena have also disappeared with the decrease of tornadoes and the increase of easterly winds. The hygrometrical observations have been so few that no comparative estimate can be made of former and latter years; yet a sufficiency is known to make certain one point, that no epidemic meteoration or any considerable prevalence of disease has ever occurred in a dry atmosphere, but are invariably accompanied by one loaded with moisture. Another fact is also developed, that a high hygrometric state of the atmosphere does not depend on the amount of rain, hence the fact that extensive disease has occurred in very wet, dry and medium seasons."

During the prevalence of epidemics, or any unusual amount of sickness, the north winds have been observed to augment the number of cases. This might be attributed to their passage over the miasmatic regions of the valley, if the same effect was not observed in connection with the easterly winds. Dr. H. reconciles this difficulty by supposing that a predisposition to autumnal disease is produced by the westerly winds, which traverse the malarious portion of the valleys of both rivers, and that the winds from the north and east act as exciting causes from other qualities that they possess besides malaria. Assuming malaria to be the cause of the fevers of this region, it is a little singular that its received sources have been steadily diminishing, whilst the diseases attributed to it are far more prevalent. And from facts in his possession, Dr. H. doubts whether the sources of miasm are correctly understood, and urges renewed inquiry.

"To identify this all-pervading influence, to give it a local habitation and a name, are efforts worthy the united energies of the medical community. And how long shall we be content to know it exists only by its effects, and rest satisfied in counteracting those effects, while we are entirely ignorant of the first principles of the cause which produces them? It has been urged, yea settled, that heat, moisture, and dead vegetable matter, will invariably produce this deleterious agent; yet how often do we find the predictions of physicians fail, even when these agents appear to be present, and under the most favorable circumstances for the generation of this aerial poison. At other times, when the temperature, moisture, and vegetable matter, would seem to indicate a state of atmosphere as pure as the mountain breeze, our pleasing anticipations of health and happiness are blasted by the deadly simoom of the autumn, which, like the wind, "bloweth where it listeth, and thou hearest the sound thereof, but canst not tell whence it cometh or whither it goeth;" at one time dealing death and destruction in the high and elevated regions of health, at others sweeping with fearful strides through the lower and more fertile but less salubrious valleys."

Among the diseases incident to this region, intermittent and remittent fevers of course hold a prominent place. These are, indeed, the only diseases to which the early settlers were liable; they usually began about the last of June and continued till the last of October, the remainder of the year being healthy. "An epidemic meteoration" has been observed five times—in 1807, '21, '22, '23, and '24. The epidemic of 1807 was very general, embracing the entire valley of the Ohio, and many of its tributaries, and involving nearly the whole population, many of whom died of the disease, or of improper treatment. This disease corresponded to the remittent fever described in the books; it appeared occasionally up to 1820. That of '21 was at first a bilious remittent fever, which after the 1st of August changed to an intermittent, and was more fatal. The epidemic of '22 was a double tertian intermittent, and prevailed generally in town and country. That of '23, was not violent and prevailed mostly in the town. That of 1824, also a double tertian, was entirely confined to the town, and was unusually severe.

Since these epidemics, the diseases of this region have greatly increased in variety, and occur throughout the year save in April and May, which are generally healthy.

Bilious pneumonia was first observed in March 1824, has since recurred every year from December to March, and was epidemic in the winter and spring of '26, '29, '31, '36, '38, '40, '41, and '42. Intermittent cephalalgia is common, at times affecting a large part of the population. Rheumatism both acute and chronic is very rare; from 1816 to 1838, Dr. H. did not see more than ten cases; since the latter period it has increased, and since 1841 has been very common. All the cases were attended by fever of the intermittent type; in many instances it was succeeded by chorea. Dr. H. relied chiefly on the alcoholic tincture of the actea racemosa, which he thinks as valuable in chorea and rheumatism, as quinine in intermittents. He likewise uses it in neuralgia, some forms of diseased liver, and in rigidity of the os uteri.

Previous to 1812 cholera infantum was unknown; after the year 1821, it became quite common, and was extremely fatal; declined after 1830, until 1841, when it was again very fatal.

Of the exanthemata, Dr. H. notices small pox, measles, hooping-cough, scarlet fever and mumps, as having prevailed at different times. These spread much more generally on the uplands than in the valley, where they seemed to lose in a great measure their contagious character. This last is also true of Asiatic cholera, which appeared in '32, and again in '33 and '34.

Croup and quinsy are uncommon—in 26 years not more than ten cases have been witnessed in the Ohio and Scioto valleys. Gangrenopsis is rather frequent, always affects decidedly scrofulous constitutions, occurs in the autumn, and generally at the close of protracted diseases. It owes its origin to the same causes that produce intermittents, and was most prevalent during the epidemic years. Phthisis pulmonalis is rare; an original case has never occurred in this locality, though scrofula in some of its forms is common.

Dr. H's paper closes with abstracts of thermometric observations from 1824 to 1835 inclusive; and of thermometric and barometric observations from 1835 to 1841. These observations were conducted by himself, and are evidently drawn up with great care. But we have already consumed a good deal of space in this notice—more perhaps than some of our readers may think warrantable; if we shall but incite to further observations of the kind, our object will be accomplished. It is only by the accumulation of labors of this sort, that medical histories of extended regions can be known; he who *asks* the *cui bono,* is not likely to understand it if he were told.

The other paper in these proceedings is on "the causes and treatment of Milksickness," by Dr. Dawson, and was prepared in obedience to a resolution of the Convention at its previous meeting. So much has appeared in the Journal on this subject, that we fear some of our readers feel a degree of nausea at the bare mention of it. We will take this opportunity, however, to express our own disbelief in any such disease. We were brought up in a region where it is believed to be present, where Dr. Seaton and others say it prevails (though the former errs in the geological formation of the country); but we never could satisfy ourselves of it. We have seen what was *called* milksickness; but it was nought else than a form of remittent fever, and was amenable to the same remedies. We have seen all the phenomena of the so called milksickness produced by worms, and cease on their expulsion. We never knew an instance that could be traced to milk, butter or beef—and we doubt very much if one ever was or ever will be. We do not deny that the beef, milk, &c., of sick cattle will produce disease in man, but we do not believe that it produces in certain districts, and no others, milksickness. Even if it did, it is made a much greater bugbear than there is any necessity for. Its prevention is easy, far more than that of most other diseases, and its attacks are not more fatal. Men rush unconcernedly into regions where the very air is thick with the elements of death, but they fly as from some horrid and wide-wasting pestilence the region where milksickness is said to prevail. Our opinion has not been formed lightly or hastily—and we believe, that if it is not now, it will be sooner or later the sentiment of the profession.

The convention was in session five days, and a large amount of business was transacted. Of the various exercises, it would be somewhat late to speak, seeing that twelve moons have passed since it adjourned. It is the less necessary too, from the notice, embracing the more important matters, given by us shortly after the adjournment.

At an early period of the sitting, those who knew the secret sources of action, who could pierce to the under-currents that flowed hither and yon, feared for the harmony of the convention. There were old feuds at work, secret heart-burnings and jealousies, wounded pride, and peradventure wounded feelings. And so new animosities were engendered by the old; factions multiplied; parties were formed; bitter words and keen retorts passed from man to man; and anger and pride were about to do their work. But a few mild words from one who had borne the toils of some forty summers and winters among that people, whose name is identified with their history, and with that of the profession in the west—words which were as true-hearted as they were few and touching—stilled the rising storm, dispelled as if by magic the ominous and surcharged clouds that were gathering for elemental play around and over-head, and then peace like a dove

> —— came forth to spy
> What calm had fallen on earth, what light was in the sky!

Explanations were made—a reconciliation took place; old injuries were mutually forgiven and forgotten; hands that erewhile were clenched in passion were now clasped in friendship—what if cheeks lately flushed with anger, were bedewed with tears from eyes all unused to them?—and now the profession in that state presents, so far as we know, an unbroken and undivided front, one in interest, purpose, and feeling. Is it so in any other State in the Union? C.

